# Validity of smartphone sensors to assess selected kinetic and kinematic outcomes during single-leg landing stabilization tasks

**DOI:** 10.1371/journal.pone.0319744

**Published:** 2025-06-03

**Authors:** Alessio Gallina, Michael Anthonius Lim, Hélio V. Cabral

**Affiliations:** 1 School of Sport, Exercise and Rehabilitation Sciences, College of Life and Environmental Sciences, University of Birmingham, Birmingham, United Kingdom; 2 Department of Clinical and Experimental Sciences, Università degli Studi di Brescia, Brescia, Italy; La Trobe University, AUSTRALIA

## Abstract

**Introduction:**

Landing stabilization tasks are a key component of injury prevention and rehabilitation programs. An objective estimation of kinetics and kinematics requires equipment and expertise which are often unavailable outside specialized centres. We investigated whether smartphone sensors, which are widely available to the general population, provide valid estimates of selected body kinematics and kinetics during landing stabilization tasks.

**Methods:**

Seventy-six asymptomatic participants (35 females) performed a unilateral landing stabilization task on both legs. Ground reaction forces and trunk orientation were collected using a force platform and an inertial measurement unit, respectively. Participants performed the tasks holding a smartphone on their chest, which collected acceleration and orientation data. We established the validity between estimates obtained with smartphone sensors and specialized equipment using Intraclass Correlation Coefficient (ICC) and paired T-test or Wilcoxon test.

**Results:**

For both legs, agreement was excellent for time of flight (lower bound of the 95% confidence interval of the ICC > 0.94); at least good for early balance (ICC > 0.87), sagittal trunk orientation (ICC > 0.79), and concentric force (ICC > 0.80); and at least moderate for frontal trunk orientation (ICC > 0.58), late balance (ICC > 0.65) and landing impact (ICC > 0.60). Smartphone accelerometers overestimated all measures compared to the force platform (p < 0.001).

**Conclusion:**

Smartphone sensors provide measures similar to those obtained using specialized equipment when used to assess selected kinetics and kinematics outcomes during landing stabilization exercises. Time of flight, concentric force, early balance and trunk orientation in the sagittal plane demonstrated at least good agreement, although smartphone sensors systematically overestimated acceleration-derived measures. This study sets the foundations for the use of smartphone sensors as a simple, inexpensive tool to characterize the performance of landing stabilization tasks objectively.

## Introduction

Landing stabilization exercises are a key component in ACL injury prevention [1,2] and rehabilitation [3]. Several kinematic and kinetic parameters during jumping, landing and stabilization have been shown to be relevant for lower limb injuries. For instance, Anterior Cruciate Ligament (ACL) injury is more likely to occur when making contact with the ground with the trunk in extension [4,5] and with larger lateral inclination [4]. Additionally, people with heavier [6] or asymmetrical [7] landing impact are more likely to sustain lower limb injuries. Some of these parameters may also be useful to identify long-lasting motor deficits after an injury. For instance, time to stabilization is longer in people with functional ankle instability [8], and individuals who have been cleared to return to sport after ACL reconstruction may still demonstrate jump height deficits on the injured side [9]. An objective assessment of body kinematics and kinetics during jumping, landing and stabilization tasks may be useful to guide exercise prescription for prevention and rehabilitation.

Kinematics and kinetics outcomes are commonly measured using laboratory-based techniques such as motion capture, inertial measurement units and force platforms. This equipment is often only available in highly specialized centres, and it requires dedicated personnel with specific technical skills, which limits its applicability to practice. While a visual assessment of body kinematics during landing may be efficient [10,11], a sensor-based assessment of motor performance may further enhance the amount and quality of information obtained. Ideally, this objective motor assessment should be performed using widely-available, inexpensive equipment.

Most modern smartphones are equipped with sensors similar to those contained in inertial measurement units. Previous research has demonstrated that these sensors are valid and reliable when measuring static range of motion [12], valid when measuring balance [13] and body acceleration [14], and reliable between days when measuring body segment orientation during dynamic tasks [15,16]. Since smartphones are widely available to the population and sensor data can be collected using free applications, smartphone sensors may be an ideal solution to characterize body kinetics and kinematics during landing stabilization tasks in practice.

In this study we investigated whether smartphone sensors can characterize body selected kinetics and kinematics outcomes during a single-leg landing stabilization task. We hypothesized that time of flight, landing impact and dynamic balance response would show at least good agreement when compared to similar measures obtained using force platforms. Similarly, we hypothesized that trunk orientation on the frontal and sagittal plane would be comparable between smartphone sensors and a research-grade inertial measurement unit.

## Materials and methods

A convenience sample of 76 adults participated in the study between March 5, 2022 and February 9, 2023. Participants were recruited by posting flyers at the institution where the study was conducted and by word of mouth, and participants needed to be 18–40 years old to be eligible to take part in the study. Exclusion criteria included lower limb or back injuries that required surgery, current lower limb or low back pain, pathologies that affect movement or balance, current or recent pregnancy, and inability to understand English. There were no limitations with respect to amount of physical activity or sport participation. This study was approved by the ethics board of the School of Sport, Exercise and Rehabilitation Sciences at the University of Birmingham (MCR2122_19), and all participants provided written informed consent before taking part in the study. Before data collection, participants self-reported anthropometrics, typical amount of time engaged in sport per week, and leg dominance. They were asked to wear comfortable clothing and trainers for the testing session, which took place in a laboratory in the School of Sport, Exercise and Rehabilitation Sciences at the University of Birmingham.

For the single-leg stabilization task, we asked the participants to stand on a force platform on one leg, jump as high as possible, land on the same leg, regain their balance as quickly as possible, and stay as still as possible for at least 5 s. We did not provide any indications with respect to trunk position. After a 5-minute self-directed warm up, participants practiced the single-leg stabilization task 5 times on each leg. Then, participants performed one set of 5 repetitions on each leg. The starting leg was randomized (55% of the participants started with the right leg). If the participant landed outside the force platform, or if they lost balance and placed their other foot on the ground, the repetition was excluded from the analysis and an additional repetition was performed. Rest between sets was not strictly monitored (usually approximately 1 minute).

Ground reaction forces (GRF) were collected at 250 Hz using a force platform embedded in the floor (BTS P6000, BTS Bioengineering, Italy). Trunk orientation and acceleration were collected at 200 Hz using a single inertial measurement unit (MyoMotion, Noraxon, USA) placed over the second thoracic vertebra. The landing stabilization tasks were performed holding a Galaxy A5 smartphone (Samsung, South Korea) in landscape orientation with the screen facing outside and the camera on the left. Participants held the smartphone just below their sternal notch. We chose to ask the participants to manually hold the smartphone because: 1) using a harness would imply the need of additional equipment, which would make the procedure more difficult to implement in practice; 2) the need to hold the smartphone limits the possibility to use the upper limbs to balance; 3) the participants were able to reposition the smartphone between trials if its position changed during the exercise; 4) it decreased the chance that the smartphone would drop when landing. We instructed the participants to apply some pressure on the smartphone to keep it in place throughout the task, and this was also by an investigator through visual inspection. Data from the smartphone acceleration and orientation sensors was collected using the Matlab Mobile app (version 5.5.0; Mathworks, Natick, USA), which sampled data at 100 Hz. The data was saved locally in the smartphone as ‘.csv’ files and exported from the smartphone using a USB cable.

**Fig 1:**
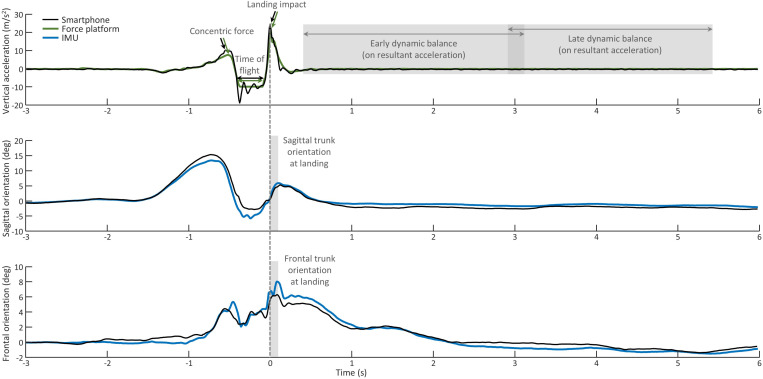
Raw data. Examples of raw data acquired from smartphone sensors (black line), force platform (green line) and inertial measurement unit (blue line). Variables extracted for each analysis are indicated in the plots. Light grey rectangles indicate time windows used for analyses. The smartphone vertical acceleration shown in the plot is that used to extract concentric force and landing impact; for the calculation of time of flight, please refer to the text. Note that early and late dynamic balance were calculated on the resultant acceleration. IMU: Inertial measurement unit.

Data was analyzed using Matlab (Mathworks, Natick, USA). Data collected with force platform, IMU and smartphone was aligned by identifying the peak GRF and the peak acceleration, which represented the landing impact. Examples of raw data and analysis are shown in Fig 1. All data was inspected visually for quality.

The acceleration values corresponding to concentric force and landing impact were extracted from: 1) the peak vertical GRF obtained from the force platform and divided by the participant’s mass calculated from the force platform data; and 2) from the vertical acceleration (X axis) in the smartphone data, adding 9.81 m/s^2^ to remove the offset due to gravity, and changing the polarity of the signal so that accelerations due to concentric force and landing would be observed as positive peaks (Fig 1). In both smartphone and IMU data, the trunk orientation at landing was calculated as the peak value in a 100 ms window starting at landing impact to represent trunk flexion and contralateral trunk flexion. To limit the effect of differences in trunk anatomy [16] and small changes in smartphone position between trials on the orientation estimates, the trunk orientation is reported as a change from baseline, measured in a 1-second window of quiet standing before each repetition of the task. In the force platform data, dynamic balance in the early (0.4 to 2.7 s after landing impact) and late (2.5 to 5 s after landing impact) phase [17] were calculated as the root mean square of the resultant force (after removing the weight from the vertical forces), similar to the dynamic postural stability index [18]. For smartphone data, balance in the same time windows was characterized using the root mean square of the resultant acceleration. In the force platform data, the time of flight was estimated as the time interval where the vertical forces were less than 10 N [19,20]. In the smartphone data, the acceleration measured on the X axis (which mainly represents vertical acceleration when the smartphone is in landscape orientation) during quiet standing was close to -9.8 m/s^2^. Since accelerometers cannot measure acceleration due to gravity while free falling (such as during a jump), vertical acceleration values close to or higher than 0 m/s^2^ indicate that the participant was in the air. To our knowledge, clear cut-off values to identify start and end of the flight time from smartphone acceleration are unknown. Therefore, we used a conservative threshold of -3 m/s^2^. This threshold was determined visually as the threshold closest to 0 m/s^2^ that was minimally affected by small fluctuations of the acceleration signal before/after take-off and landing. We also confirmed this by analyzing our data with thresholds between 0 and -5 m/s^2^ (S1 Table), demonstrating excellent validity when the threshold was -2 m/s^2^ or lower. We therefore estimated the time of flight from smartphone acceleration data as the time interval between the timestamp of the first sample when acceleration exceeded -3 m/s^2^, to the timestamp of the last sample above -3 m/s^2^ before the landing impact. For all outcomes, the median value across 5 repetitions was calculated for the dominant and for the non-dominant leg and used for the analyses.

Statistical analyses were performed using SPSS (v 28.0.1.1; IBM, Armonk, USA). We used the Shapiro-Wilk test to establish whether the data was normally distributed. Based on the data distribution, for each outcome we used paired T-test or Wilcoxon paired sample test to determine whether there was a systematic bias between measures obtained with smartphone and laboratory equipment or between legs (supplementary material). We determined the agreement between measures visually using Bland-Altman plots, and formally using two-way, random model Intraclass Correlation Coefficients (ICC) for average measures. If a systematic bias was observed, we used an ICC model for consistency; otherwise, the ICC estimates refer to absolute agreement. The lower bound of the ICC was used to classify the agreement as excellent (>0.90), good (>0.75), moderate (>0.5), poor (<0.5) [21]. Data are reported as mean (standard deviation) or median [25^th^, 75^th^ percentiles] depending on the data distribution, and the significance level was set at 0.05. All the individual data values are provided in the supplementary materials.

## Results

Participants were 22.3 ± 3.8 (range: 18–34) year old, 35 were females, and the average height and weight were 172.8 ± 9.4 cm and 70.8 ± 12.9 kg. Most participants were students at the institution where the study took place, and they self-reported to engage in sports for 3 [1, 4] hours per week. Seven participants reported their left leg as dominant. Smartphone orientation data was not collected from 11 participants because the sensor was not activated during the data collection. Smartphone data was not saved correctly for one participant when performing the task on one leg. These participants were excluded from the analyses for which no data was available, but they were retained for the other analyses.

**Fig 2:**
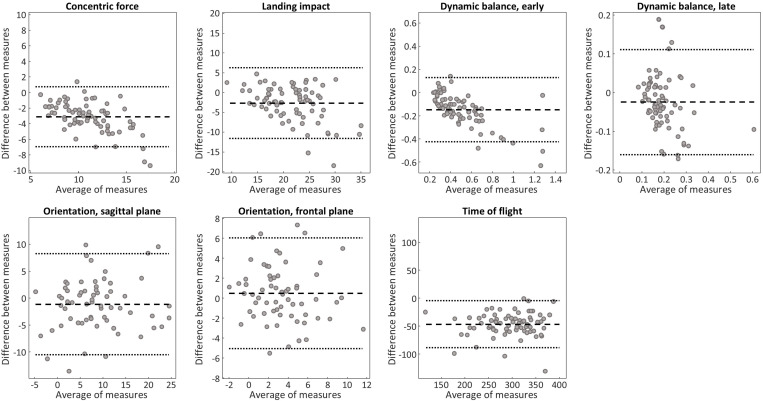
Validity analysis. Bland-Altman plots between acceleration estimates from smartphone and force platform (time of flight, concentric force, landing impact, early and late dynamic balance), and between orientation estimates from smartphone and inertial measurement unit (sagittal and frontal trunk orientation).

Systematic bias between measures and agreement can be observed in the Bland-Altman plots in Fig 2 and Table 1, which also shows the number of participants included in each statistical test. Point ICC estimates ranged between 0.74 (trunk orientation in the frontal plane) to 0.97 (time of flight). Based on the lower bound of the 95% confidence interval of the ICC, agreement was: excellent for time of flight; at least good for balance in the early dynamic phase, concentric force, sagittal trunk orientation; and at least moderate for: frontal trunk orientation, balance in the late dynamic phase, and landing impact (Table 1). The Bland-Altman plots show trends for larger errors when concentric force and landing impacts are larger, and in participants with larger postural sways in the early dynamic phase of balance (Fig 2). Smartphone sensors consistently overestimated acceleration-based measures (all p < 0.001), whereas trunk orientation on the frontal plane when landing on the non-dominant leg demonstrated a systematic bias (p < 0.001; Table 1). Validity assessed for left and right leg (S2 Table) was similar to that observed for dominant and non-dominant leg (Table 1). When comparing between left and right, smartphone sensors did not identify any differences in performance between sides, whereas laboratory instrumentation identified worse balance when landing on the right leg and more side trunk flexion when landing on the left leg (S3 Table).

**Table 1:** Validity of smartphone sensors compared to force platform and inertial measurement units.

OUTCOME	SIDE	N	FORCE PLATFORM	SMARTPHONE	BIAS	ICC
Time of flight	ND	76	276 [228, 312]	327 [275, 354]	**p < 0.001**	0.97 [0.95, 0.98]^c^
	D	75	280 [236, 314]	325 [285, 355]	**p < 0.001**	0.96 [0.94, 0.98]^c^
Concentric force	ND	76	9.2 [7.5, 11.4]	11.8 [9.7, 14.7]	**p < 0.001**	0.91 [0.86, 0.94]^c^
	D	75	9.6 [7.8, 10.9]	12.1 [10.1, 14.6]	**p < 0.001**	0.87 [0.80, 0.92]^c^
Landing impact	ND	76	20.3 (4.9)	23.0 (6.8)	**p < 0.001**	0.75 [0.60, 0.84]^c^
	D	75	20.2 (4.7)	22.9 (6.5)	**p < 0.001**	0.81 [0.70, 0.88]^c^
Balance, early	ND	76	0.34 [0.28, 0.46]	0.44 [0.34, 0.67]	**p < 0.001**	0.94 [0.91, 0.97]^c^
	D	75	0.38 [0.31, 0.54]	0.51 [0.36, 0.71]	**p < 0.001**	0.92 [0.87, 0.95]^c^
Balance, late	ND	76	0.16 [0.13, 0.18]	0.17 [0.14, 0.24]	**p < 0.001**	0.78 [0.65, 0.86]^c^
	D	75	0.16 [0.14, 0.21]	0.19 [0.15, 0.25]	**p < 0.001**	0.78 [0.65, 0.86]^c^
OUTCOME	**SIDE**		**IMU**	**SMARTPHONE**	**BIAS**	**ICC**
Sagittal trunk orientation	ND	65	6.4 [2.0, 9.9]	6.5 [2.6, 12.1]	p = 0.115	0.89 [0.81, 0.93]^a^
	D	64	7.2 [2.6, 11.1]	8.0 [3.6, 12.7]	p = 0.051	0.88 [0.79, 0.92]^a^
Frontal trunk orientation	ND	65	4.8 (3.9)	2.5 (3.7)	**p < 0.001**	0.82 [0.70, 0.89]^c^
	D	64	3.5 (3.1)	3.1 (3.3)	p = 0.171	0.74 [0.58, 0.84]^a^

D, dominant; ND, non-dominant; IMU, inertial measurement unit; ICC, intraclass correlation coefficient, with 95% confidence intervals. a: ICC for absolute agreement; c: ICC for consistency.

## Discussion

Smartphone sensors have moderate-to-excellent validity compared to specialized research equipment when used to assess selected kinetic and kinematic outcomes during single-leg landing stabilization tasks. Based on the lower bound of the intraclass correlation coefficient, agreement was at least good for time of flight, concentric force, balance in the early dynamic phase and trunk orientation on the sagittal plane; the other outcomes demonstrated at least moderate agreement. Acceleration-based measures demonstrated a systematic bias between smartphone sensors and specialized research equipment. The potential to use smartphone sensors to characterize the performance of landing stabilization tasks in practice and research should be explored further.

Our results concur with other research supporting the validity of smartphone sensors to measure body segment orientation in static tasks [12] and balance [13], and the use of accelerometers to assess jump height [22–24] and landing impacts [22,25]. The variability in the observed range of agreement and bias estimates may be explained by different reasons. The late dynamic balance phase demonstrated lower agreement than early dynamic balance, possibly because the smaller body sways resulted in lower variance between participants. Smartphones were also unable to detect side differences in balance identified by force platform measures, possibly because of the small difference. Landing impact demonstrated lower agreement than concentric force, possibly because of some variability introduced by small movements of the smartphone with respect to the chest due to the landing impact, given that acceleration readings from inertial devices are highly sensitive to orientation. Similarly, small movements of the smartphone with respect to the chest due to the landing impact may have contributed to only moderate-to-good agreement of the orientation measures; lower agreement for frontal compared to sagittal plan are likely due to a smaller range of values for frontal plane estimates. Since previous research demonstrated that the ability of smartphone sensors to detect differences in balance performance across exercises depends on where the smartphone is positioned [22], different smartphone locations may result in better agreement. With respect to the bias analysis, smartphones overestimated all acceleration magnitude measures compared to force platforms. Orientation estimates only demonstrated significant bias on the frontal plane when landing on the non-dominant leg, with smartphones measuring about 1.5 degree less contralateral inclination than the IMU, and failing to detect side-differences identified by the laboratory equipment (S3 Table). It is possible that performing the task on the non-dominant leg was more attention-demanding, and therefore participants were less careful in maintaining the smartphone aligned on their chest when landing. Finally, despite almost perfect agreement, smartphones significantly overestimated time of flight compared to force platforms. Although there is evidence that accelerometers overestimate jump height compared to force plates [23,24], our choice of threshold may also have contributed to the large bias observed; while less conservative thresholds (closer to 0 m/s^2^) may reduce this bias, they also result in lower agreement (S1 Table) possibly because of an increased sensitivity to small changes in acceleration due changes in body orientation. For all the outcomes that demonstrated a significant bias, the bias should be considered when comparing outcomes estimated from force platforms and smartphones. Similarly, the trends observed in the Bland-Altman plots suggest that the overestimation of early dynamic balance, concentric force, and landing impact is more significant for larger postural sways, larger concentric forces and heavier impacts.

The approach proposed has some limitations which should be addressed in future studies. First, we only used a single smartphone; since orientation [26] and acceleration [27] estimates have been shown to differ for smartphones of different brands and models, it should be investigated whether these differences are large enough to impact the validity observed in this study. Similarly, the between-day reliability should be investigated before smartphone sensors are used to monitor changes in performance over time. Data was collected under supervision of researchers, and participants manually hold the smartphone; it is unclear if the measures would have similar validity if participants collected data on their own or securing the smartphone using a harness or an elastic band. We did not collect orientation data from 11 participants because the sensor was not activated during data collection; a more user-friendly application that automatically enables the sensors necessary for the test would decrease the chance of missing data. Finally, data was analyzed using custom-written code; in-app or automatized data visualization and analysis procedures would help implement this procedure in practice.

## Perspectives

This work sets the foundations for a simple and inexpensive way to objectively estimate multiple kinematic and kinetic variables that have been shown to be relevant for performance, injury prevention and rehabilitation [6,8,9]. Since smartphones are widely available to the general population and there are several other free applications available besides Matlab Mobile (e.g.,: Phyphox, [28]), data collection with smartphone sensors is more easily accessible than data collection with specialized equipment, and can be easily performed remotely. We observed overall high validity even when participants held the smartphone manually, implying that valid data can be collected without the need of additional equipment such as a harness, which may not be readily available in practice. Future work should improve accessibility of this methodology to students, researchers and clinicians [29], and establish whether the use of smartphone sensors to monitor physical performance over time improves the effectiveness of injury prevention and rehabilitation interventions.

## Conclusions

Compared to specialized research equipment, smartphone sensors provide valid estimates of time of flight, concentric force, balance in the early dynamic phase and trunk orientation during landing stabilization tasks, although they systematically overestimated acceleration-derived measures. Smartphone sensors have the potential to be used as an inexpensive, easily accessible tool to objectively characterize selected kinetic and kinematic outcomes during landing stabilization tasks.

## Supporting information

Table S1 Effect of threshold on the validity of the Time of Flight estimation. (DOCX)

Table S2 Validity of smartphone sensors compared to force platform and inertial measurement units for left and right side. (DOCX)

Table S3 Comparison between left and right side. (DOCX)
