# Supplementary material for: Validity of smartphone sensors to assess selected kinetic and kinematic outcomes during single-leg landing stabilization tasks
Source: PLoS One. 2025 Jun 3;20(6):e0319744. doi: 10.1371/journal.pone.0319744 (PMC12133011; doi:10.1371/journal.pone.0319744)
Supplement: Table S1 — (DOCX) [file pone.0319744.s001.docx]

**Table S1. Effect of threshold on the validity of the Time of Flight estimation.** Threshold indicates the acceleration value used to define take-off and landing to estimate the time of flight.

| **THRESHOLD** | **TIME OF FLIGHT (SMARTPHONE)** | **BIAS** | **ICC** |
| --- | --- | --- | --- |
| **0** | 268 [217, 312] | p=0.003 | 0.83 [0.74, 0.90] |
| **-1** | 306 [256, 336] | p=0.005 | 0.93 [0.88, 0.95] |
| **-2** | 316 [276, 350] | p<0.001 | 0.96 [0.93, 0.97] |
| **-3** | 325 [285, 355] | p<0.001 | 0.96 [0.94, 0.98] |
| **-4** | 336 [292, 364] | p<0.001 | 0.96 [0.93, 0.97] |
| **-5** | 346 [305, 373] | p<0.001 | 0.96 [0.93, 0.97] |

*Intraclass Correlation Coefficient, with 95% confidence intervals.*
