# Supplementary material for: Validity of smartphone sensors to assess selected kinetic and kinematic outcomes during single-leg landing stabilization tasks
Source: PLoS One. 2025 Jun 3;20(6):e0319744. doi: 10.1371/journal.pone.0319744 (PMC12133011; doi:10.1371/journal.pone.0319744)
Supplement: Table S2 — (DOCX) [file pone.0319744.s002.docx]

**Table S2. Validity of smartphone sensors compared to force platform and inertial measurement units for left and right side.**

| **OUTCOME** | **SIDE** | **N** | **FORCE**  **PLATFORM** | **SMARTPHONE** | **BIAS** | **ICC** |
| --- | --- | --- | --- | --- | --- | --- |
| **Time of flight** | L | 76 | 276 [228, 313] | 326 [275, 356] | **p<0.001** | 0.97 [0.96, 0.98]^c^ |
|  | R | 75 | 280 [236, 310] | 325 [285, 355] | **p<0.001** | 0.96 [0.93, 0.97]^c^ |
| **Concentric force** | L | 76 | 9.2 [7.5, 11.4] | 12.1 [9.9, 14.7] | **p<0.001** | 0.91 [0.85, 0.94]^c^ |
|  | R | 75 | 9.2 [7.8, 11.0] | 12.0 [9.9, 14.4] | **p<0.001** | 0.88 [0.81, 0.92]^c^ |
| **Landing impact** | L | 76 | 20.3 (5.1) | 23.0 (6.9) | **p<0.001** | 0.77 [0.63, 0.85]^c^ |
|  | R | 75 | 20.2 (4.5) | 22.8 (6.5) | **p<0.001** | 0.79 [0.67, 0.87]^c^ |
| **Balance, early** | L | 76 | 0.34 [0.28, 0.46] | 0.45 [0.34, 0.67] | **p<0.001** | 0.94 [0.91, 0.96]^c^ |
|  | R | 75 | 0.38 [0.30, 0.54] | 0.51 [0.36, 0.71] | **p<0.001** | 0.92 [0.88, 0.95]^c^ |
| **Balance, late** | L | 76 | 0.15 [0.12, 0.18] | 0.18 [0.13, 0.24] | **p<0.001** | 0.71 [0.55, 0.82]^c^ |
|  | R | 75 | 0.17 [0.14, 0.21] | 0.19 [0.15, 0.25] | **p=0.003** | 0.82 [0.71, 0.86]^c^ |
|  | | | | | | |
| **OUTCOME** | **SIDE** | **N** | **IMU** | **SMARTPHONE** | **BIAS** | **ICC** |
| **Sagittal trunk orientation** | L | 65 | 6.6 [2.0, 10.5] | 6.7 [2.6, 13.2] | p=0.068 | 0.90 [0.83, 0.94]^a^ |
|  | R | 64 | 7.2 [2.6, 11.0] | 8.1 [3.6, 11.5] | p=0.081 | 0.86 [0.77, 0.92]^a^ |
| **Frontal trunk orientation** | L | 65 | 5.0 (3.9) | 2.6 (3.8) | **p<0.001** | 0.82 [0.71, 0.89]^c^ |
|  | R | 64 | 3.3 (3.0) | 3.1 (3.2) | p=0.342 | 0.75 [0.59, 0.85]^a^ |

*L: Left; R: Right; IMU: Inertial Measurement Unit; ICC: Intraclass Correlation Coefficient, with 95% confidence intervals.*
