# Supplementary material for: Validity of smartphone sensors to assess selected kinetic and kinematic outcomes during single-leg landing stabilization tasks
Source: PLoS One. 2025 Jun 3;20(6):e0319744. doi: 10.1371/journal.pone.0319744 (PMC12133011; doi:10.1371/journal.pone.0319744)
Supplement: Table S3 — (DOCX) [file pone.0319744.s003.docx]

**Table S3. Comparison between left and right side.**

| **OUTCOME** | **MEASURE** | **N** | **LEFT** | **RIGHT** | **SIDE DIFFERENCE** |
| --- | --- | --- | --- | --- | --- |
| **Time of flight** | FP | 75 | 276 [228, 313] | 280 [236, 310] | p=0.284 |
|  | SM | 75 | 326 [275, 356] | 325 [285, 355] | p=0.246 |
| **Concentric force** | FP | 75 | 9.2 [7.5, 11.4] | 9.2 [7.8, 11.0] | p=0.212 |
|  | SM | 75 | 12.1 [9.9, 14.7] | 12.0 [9.9, 14.4] | p=0.767 |
| **Landing impact** | FP | 75 | 20.3 (5.1) | 20.2 (4.5) | p=0.908 |
|  | SM | 75 | 23.0 (6.9) | 22.8 (6.5) | p=0.590 |
| **Balance, early** | FP | 75 | 0.34 [0.28, 0.46] | 0.38 [0.30, 0.54] | p=0.138 |
|  | SM | 75 | 0.45 [0.34, 0.67] | 0.51 [0.36, 0.71] | p=0.113 |
| **Balance, late** | FP | 75 | 0.15 [0.12, 0.18] | 0.17 [0.14, 0.21] | **p<0.001** |
|  | SM | 75 | 0.18 [0.13, 0.24] | 0.19 [0.15, 0.25] | p=0.094 |
|  | | | | | |
| **OUTCOME** | **TOOL** | **N** | **LEFT** | **RIGHT** | **BIAS** |
| **Sagittal trunk orientation** | IMU | 75 | 6.6 [2.0, 10.5] | 7.2 [2.6, 11.0] | p=0.222 |
|  | SM | 64 | 6.7 [2.6, 13.2] | 8.1 [3.6, 11.5] | p=0.297 |
| **Frontal trunk orientation** | IMU | 75 | 5.0 (3.9) | 3.3 (3.0) | **p=0.003** |
|  | SM | 64 | 2.6 (3.8) | 3.1 (3.2) | p=0.448 |

*SM: Smartphone; FP: Force plate; IMU: Inertial Measurement Unit.*
